# Hidden reservoir of highly adaptable multi-host plasmids that propagate antibiotic genes in healthy human populations

**DOI:** 10.1093/ismejo/wrag004

**Published:** 2026-01-23

**Authors:** Na Han, Xianhui Peng, Tingting Zhang, Yujun Qiang, Xiuwen Li, Wen Zhang

**Affiliations:** National Key Laboratory of Intelligent Tracking and Forecasting for Infectious Diseases, National Institute for Communicable Disease Control and Prevention, Chinese Center for Disease Control and Prevention, Beijing 102206, China; National Key Laboratory of Intelligent Tracking and Forecasting for Infectious Diseases, National Institute for Communicable Disease Control and Prevention, Chinese Center for Disease Control and Prevention, Beijing 102206, China; National Key Laboratory of Intelligent Tracking and Forecasting for Infectious Diseases, National Institute for Communicable Disease Control and Prevention, Chinese Center for Disease Control and Prevention, Beijing 102206, China; National Key Laboratory of Intelligent Tracking and Forecasting for Infectious Diseases, National Institute for Communicable Disease Control and Prevention, Chinese Center for Disease Control and Prevention, Beijing 102206, China; National Key Laboratory of Intelligent Tracking and Forecasting for Infectious Diseases, National Institute for Communicable Disease Control and Prevention, Chinese Center for Disease Control and Prevention, Beijing 102206, China; National Key Laboratory of Intelligent Tracking and Forecasting for Infectious Diseases, National Institute for Communicable Disease Control and Prevention, Chinese Center for Disease Control and Prevention, Beijing 102206, China

**Keywords:** plasmidome, antibiotic resistance, pGut1, horizontal gene transfer, gut microbiome, mobile genetic elements, metagenomics, *Salmonella enterica*, stealth plasmid

## Abstract

Plasmids are key vectors for disseminating antibiotic resistance genes, yet their diversity and dynamics in the healthy human gut microbiome remain largely unexplored. Using fecal metagenomes from two cohorts (n = 498 samples), we constructed a comprehensive atlas of the healthy human gut plasmidome. We observed a polarization: while 97.4% of 19 151 plasmid clusters exhibited low prevalence (<5%), we identified 17 plasmid clusters that were detected in >30% of individuals. Among these, the plasmid pGut1 emerged as a paradigm of a stealth vector. Prevalent globally (>50% in independent cohorts), pGut1 possesses a minimal 4-kb conserved backbone ensuring stability and a hypervariable region acting as a “plug-and-play” module. We documented 40 distinct cargo inserts, including multiple antibiotic resistance genes such as *cfr(C), erm(B)*, and *aphA*, across individuals, within individuals over time, and even within single fecal samples- validated by single-cell and long-read Nanopore sequencing. Screening of 2.3 million bacterial genomes revealed pGut1 in 93 strains across 49 genera and 2 phyla, including pathogenic *Clostridioides difficile* and three distinct *Salmonella enterica* strains. This pattern suggests potential repeated cross-species transmission events, equipping diverse pathogens with new antibiotic resistance genes. Our study exposes a hidden reservoir of highly adaptable, multi-host plasmids like pGut1 silently propagating antibiotic resistance genes in healthy populations. These plasmids, pre-adapted for cross-boundary dissemination, may pose a threat by fueling the emergence of multidrug-resistant pathogens.

The healthy human gut microbiome is a vast reservoir of antibiotic resistance genes (ARGs), with plasmids serving as primary vectors for their horizontal transfer [1–4]. However, the comprehensive landscape of the gut plasmidome remains obscured by technical challenges in metagenomic assembly and database biases toward cultured clinical isolates [5–7]. To address this gap, we analyzed fecal metagenomes from two cohorts—a cross-sectional cohort (CMP_region, n = 258) and a longitudinal cohort (CMP_multitime, n = 240) (Fig. S1A)—to characterize the plasmid diversity in healthy individuals.

We identified 23,360 plasmid clusters (PLCs) from metagenome-assembled genomes, with only 6.4% showing high similarity to known plasmids in the plasmid database (PLSDB, https://ccb-microbe.cs.uni-saarland.de/plsdb2025/) [8] (Fig. S1B), highlighting a vast unexplored plasmid diversity (Fig. S1C and D). Analysis revealed extreme polarization in plasmid prevalence: 97.4% of PLCs were rare (<5% prevalence), while a small subset of 17 PLCs were detected in >30% of individuals (Fig. S1F). We term these high-prevalence, previously undetected elements “stealth plasmids”, as they have remained undetected due to technical limitations in conventional metagenomic analyses (Fig. S1F). These high-prevalence PLCs demonstrated significantly longer persistence in the longitudinal cohort compared to low-prevalence PLCs (mean 45.13 vs. 22.50 months; Wilcoxon-test, *P* < 0.05) (Fig. S1E), suggesting enhanced colonization fitness. Among ARG-carrying plasmids (0.8% of all PLCs), we observed plasticity in gene carriage. Individual PLCs exhibited context-dependent cargo flexibility, carrying different ARGs across hosts or ecological contexts (Fig. S2).

Among 17 “stealth plasmids” identified in this study, pGut1 (Accession ID: NMDCN000A675) was conspicuous for its dominance, being detected in 96.5% of samples. Its prevalence significantly exceeded that of the previously characterized *pBI143* plasmid [5] (ranked 11th in our study), establishing pGut1 as a dominant yet previously overlooked member of the “stealth plasmid” group. To comprehensively evaluate the geographical and population distribution of pGut1*,* we analyzed 148 healthy individuals from the US Human Microbiome Project (HMP) [9], detecting pGut1 in 79 samples (53.4%). This finding confirms its widespread occurrence across geographically distinct populations. The difference in detection rates between our Chinese cohort (96.5%) and the HMP cohort (53.4%) may likely reflect geographical variations, dietary influences, or methodological differences in sample processing and sequencing protocols.

Through a combination of PCR amplification and Sanger sequencing, we resolved the complete pGut1 sequence. pGut1 possesses a compact, conserved backbone (~4006 bp) encoding essential genetic elements (Fig. 1A): a replication protein, a recombinase, and a YafQ-RelB toxin-antitoxin (TA) stabilization system. The backbone sequences displayed highly conservation across positive samples (average nucleotide p-distance = 0.00069). In contrast, a large hypervariable region exhibited extensive genetic diversity, harboring 40 distinct cargo insertions identified across individuals. These insertions included multiple ARGs (e.g., *cfr(C), erm(B), aphA, linA*) and various other functional genes.

**Figure 1:**
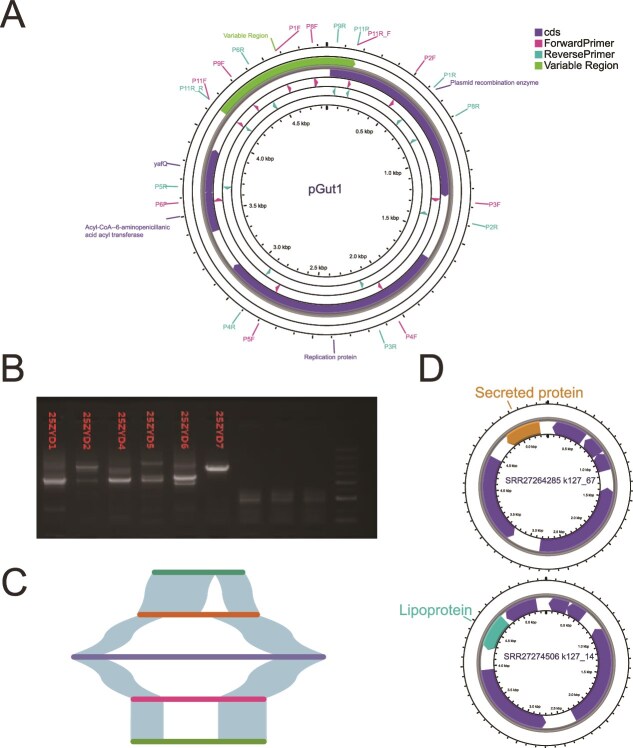
Structural and functional features of the pGut1 plasmid. (A) Circular representation of pGut1. The variable region is highlighted in green. (B) PCR validation of pGut1 across six samples using primers pGut1_P11 and pGut1_P11_reverse. (C) Structural variation among five pGut1 variant regions reconstructed from nanopore long-read sequencing of sample 20ZYI1. Connecting lines indicate sequence identity between variants. (D) Comparison of two pGut1 plasmids obtained through single-cell sequencing from individual bacterial genomes (SRR27264285 and SRR27274506) within the same sample. Their variable regions encoding distinct putative proteins: a secreted protein (yellow arrow) and a lipoprotein (teal arrow).

The structural plasticity of pGut1 manifested across multiple biological scales. Longitudinal analysis revealed dynamic insertional polymorphism within individuals. For instance, in participant P4, pGut1 was detected in both August and September 2017 samples, with the September variant uniquely acquiring an additional *erm* gene within its variable region. Similarly, in participant P2, pGut1 maintained persistence across multiple timepoints but exclusively carried the *pnuC* gene in the January 2022 sample.

This plasmid heterogeneity extended to the single-sample level, where multiple pGut1 variants carrying divergent inserts coexisting within individual fecal specimens. We validated this intra-sample diversity through three orthogonal approaches: First, PCR amplification targeting the variable region yielded multiple distinct amplicon sizes in single samples (Fig. 1B), suggesting structure variation, though potential non-specific amplification required further verification. Second, Nanopore long-read sequencing of pGut1-positive sample 20ZYI1 generated 14 complete pGut1 sequence from 4 785 475 long reads (Avg length 1004 bp), which resolved into five structurally distinct variants carrying unique insertion profiles (Fig. 1C). Third, to confirm variant coexistence at cellular resolution, we reanalyzed single-cell sequencing data (PRJNA803937) from a healthy stool sample. Among 1995 assembled bacterial genomes in a sample, two discrete genomes (SRR27264285 and SRR27274506) each harbored a pGut1 plasmid with functionally divergent variable regions—one encoding a secreted protein and the other a lipoprotein in their variable regions, respectively (Fig. 1D). This profound intra-sample heterogeneity presented significant technical assembly challenges: the coexistence of multiple variable sequences generated complex repeat structures that prevented complete plasmid circularization of pGut1 in short-read metagenomic assemblies. This technical limitation likely contributed to pGut1’s historical obscurity despite its high prevalence. Our multi-platform analytical approach—combining PCR fragment analysis, long-read sequencing, and single-cell genomics—successfully overcame these obstacles, enabling the first comprehensive characterization of these elusive plasmids, and revealing unprecedented plasmid diversity within individual microbiomes.

To investigate the phylogenetic distribution and host range of pGut1, we conducted a comprehensive screen of 2,299,771 publicly available bacterial genomes retrieved from the NCBI genome database (accessed January 2025). pGut1 was identified in 217 genomes. Following filtering to exclude potentially chimeric metagenome-assembled genomes (MAGs), we confirmed its presence in 93 high-quality genomes spanning 53 species, 49 genera, and 2 phyla (Fig. 2A). This broad host range may contribute to the high positive rate of pGut1 observed in the human gut. Phylogenetic analysis based on conserved backbone revealed clustering of closely related pGut1 variants across diverse hosts (Fig. 2A), providing clues for candidate recent cross-species horizontal gene transfer (HGT) events. For instance, two distinct strains of *Enterocloster bolteae* were found to harbor pGut1 variants, each exhibiting the highest sequence similarity to pGut1 plasmids from different species-*Ruthenibacterium lactatiformans* and *Flavonifractor plautii*, respectively, suggesting potential independent inter-species plasmid transfer events.

**Figure 2:**
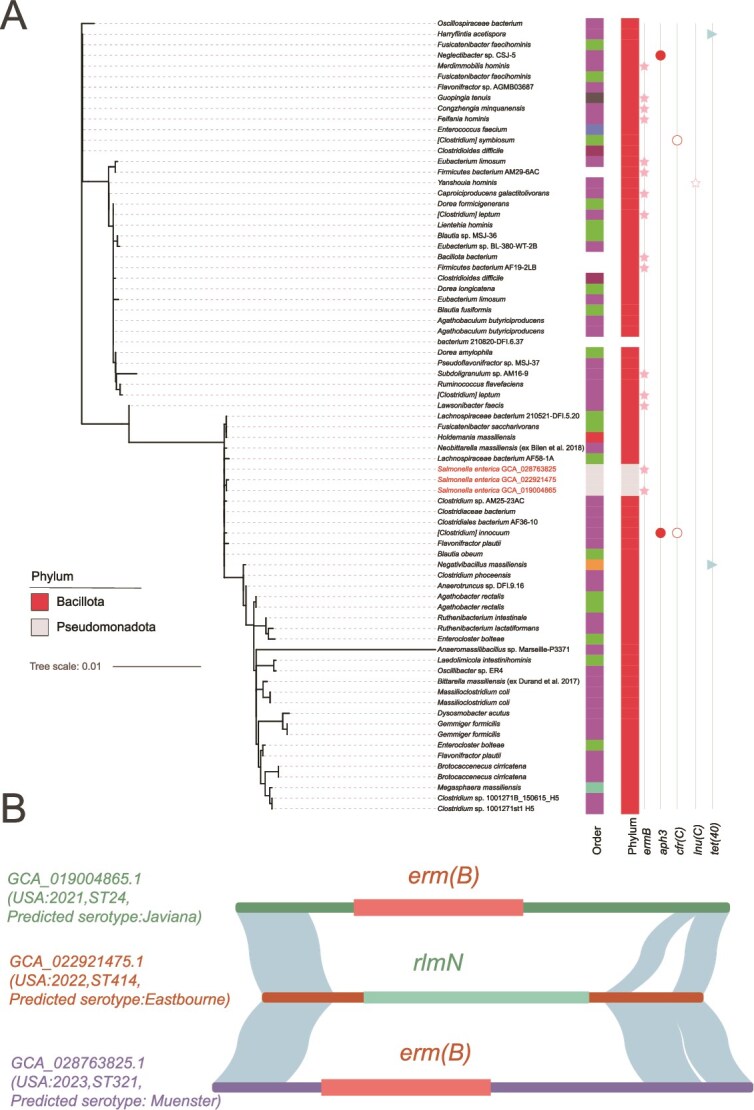
Distribution and genetic features of pGut1 across public bacterial genomes. (A) Phylogenetic tree constructed from pGut1 sequences identified in 93 high-quality bacterial genomes spanning 53 species, 49 genera, and 2 phyla. Right markers indicate host taxonomy (order and phylum), and the presence of five antibiotic resistance genes (*Erm(B)*, *aph(3′)*, *cfr(C)*, *lnu(C)*, and *tet(40)*). Three *salmonella enterica* strains are highlighted in red. (B) Linear maps of pGut1 variants from three *salmonella enterica* strains (submitted by the US CDC between 2021 and 2023). Two variantscarry an *erm(B)* insertion in their variable regions, while the third harbors an *rlmN* gene.

Host analysis revealed 95.7% of pGut1-positive isolates belonged to the *Bacillota* phylum, predominantly comprising gut commensals, and anaerobes. However, we identified pGut1 in three strains of the pathogenic Gram-negative bacterium *S. enterica* (submitted by the US CDC in 2021–2023) (Fig. 2B). These strains possessed different Multi-Locus Sequence Types (MLST) and serotypes, indicating multiple independent acquisition events. Their pGut1 variants carried different inserts: two harbored the *erm(B)* gene, whereas the third carried *rlmN* (Fig. 2B). This supports repeated cross-species transfer (even potentially cross-phylum transfer, from Bacillota to Pseudomonadota) of pGut1 from commensal reservoirs into a clinical pathogen, with potential implications for antimicrobial resistance dissemination.

Our study establishes pGut1 as a paradigm for stealth plasmid-mediated resistance transmission. Its combination of high prevalence, genetic promiscuity, and multi-host colonization capacity creates a potentially dangerous vector for incubating pan-resistant infections. Collectively, these findings reveal that the healthy gut plasmidome contains highly adaptable, pre-adapted vectors that silently propagate ARGs, and are poised for cross-boundary dissemination into pathogens. Integrating plasmid-centric metagenomic surveillance into existing AMR monitoring programs is therefore crucial to intercept such emerging threats before they contribute to outbreaks.

## Supplementary Material

SFig1_wrag004

SFig2_wrag004

Supplemental_Method_wrag004

## Data Availability

All raw sequencing reads have been deposited in the NMDC Nucleotide database (pGut1 Accession ID: NMDCN000A675) and NCBI SRA database (SAMN44930011-SAMN44930200, SAMN41553299-SAMN41553537). The bioinformatics pipeline is available on GitHub (https://github.com/zhangwencdc/pGut).
