# Supplementary material for: Hidden reservoir of highly adaptable multi-host plasmids that propagate antibiotic genes in healthy human populations
Source: ISME J. 2026 Jan 23;20(1):wrag004. doi: 10.1093/ismejo/wrag004 (PMC12919442; doi:10.1093/ismejo/wrag004)
Supplement: SFig1_wrag004 [file sfig1_wrag004.pdf]

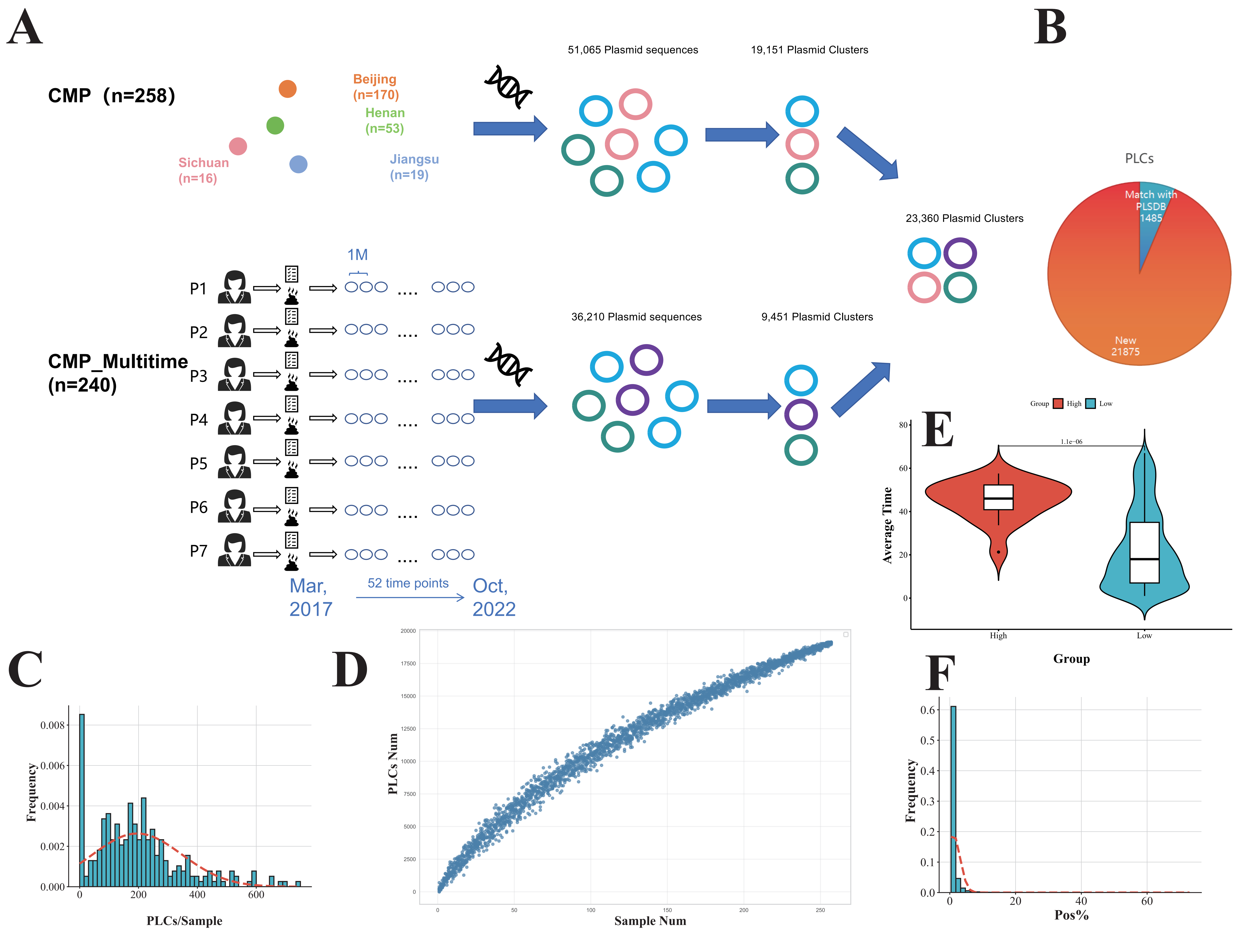
A
B
CMP (n=258)
Beijing (n=170)
Henan (n=53)
Jiangsu (n=19)
Sichuan (n=16)
51,065 Plasmid sequences
19,151 Plasmid Clusters
23,360 Plasmid Clusters
PLCs
Match with PLSDB 1485
New 21875
CMP_Multitime (n=240)
P1
P2
P3
P4
P5
P6
P7
1M
36,210 Plasmid sequences
9,451 Plasmid Clusters
Mar, 2017
52 time points
Oct, 2022
E
Group High Low
1.1e-06
Average Time
Group
C
Frequency
PLCs/Sample
D
PLCs Num
Sample Num
F
Frequency
Pos%
