# Supplementary figures and images for: Hidden reservoir of highly adaptable multi-host plasmids that propagate antibiotic genes in healthy human populations
Source: ISME J. 2026 Jan 23;20(1):wrag004. doi: 10.1093/ismejo/wrag004 (PMC12919442; doi:10.1093/ismejo/wrag004)

A

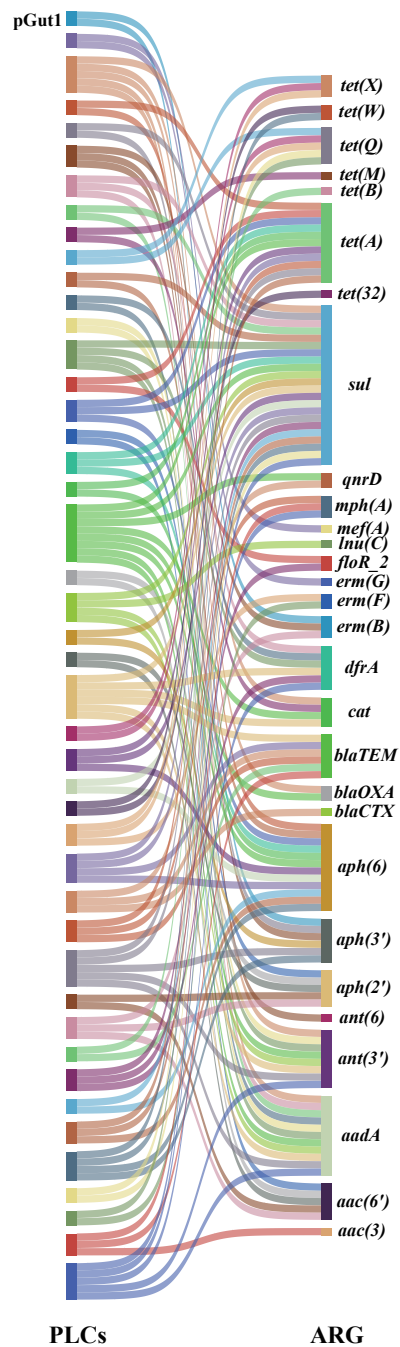

B

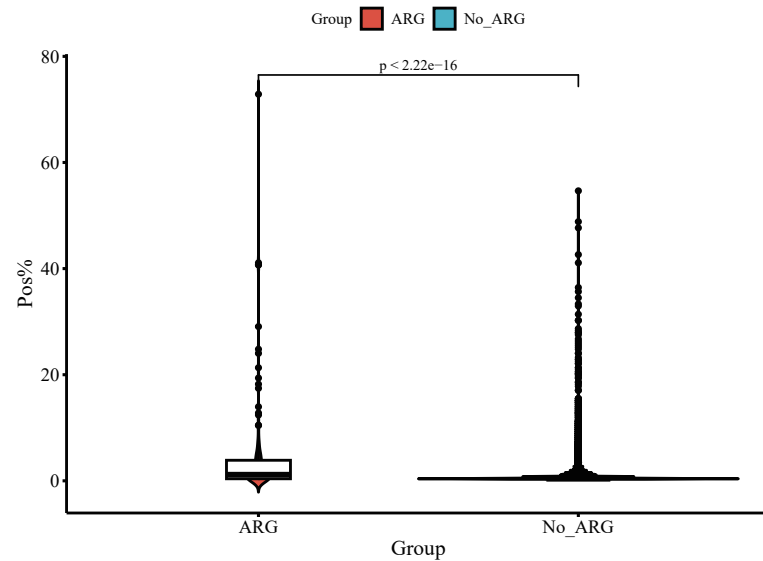

C

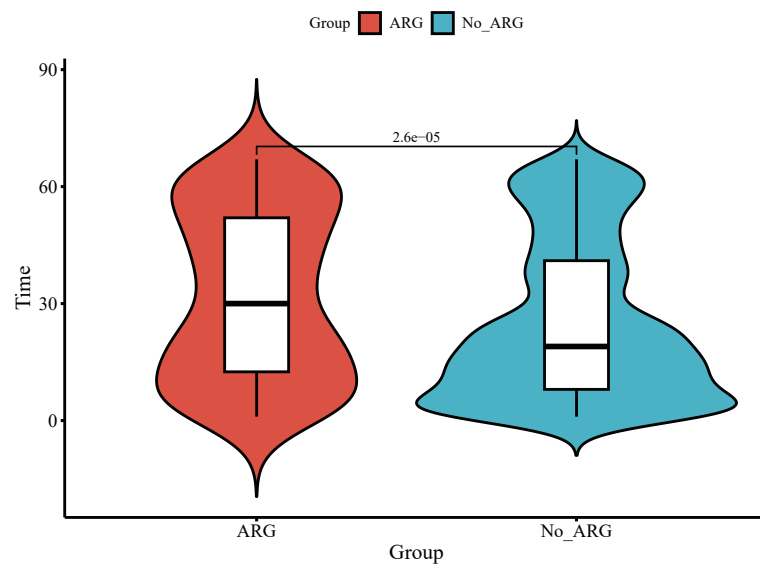

Supplement: SFig2_wrag004 [file sfig2_wrag004.pdf]
