# Supplementary material for: Hidden reservoir of highly adaptable multi-host plasmids that propagate antibiotic genes in healthy human populations
Source: ISME J. 2026 Jan 23;20(1):wrag004. doi: 10.1093/ismejo/wrag004 (PMC12919442; doi:10.1093/ismejo/wrag004)
Supplement: Supplemental_Method_wrag004 [file supplemental_method_wrag004.pdf]

Method

1 Study design and sample collection protocol for the CMP cohort

We prospectively established two independent cohorts (SFig 1A):

CMP_region cohort: Fecal samples were obtained from 258 healthy volunteers (age range: 18-70, BMI 16-30 kg/m^2) across four Chinese provinces (Beijing, Jiangsu, Henan, Sichuan)(Zhang et al 2019b).

CMP_MultiTime cohort: A total of 240 longitudinal fecal samples were collected from 7 volunteers at 52 timepoints (March 2017–October 2022)(Zhang et al 2022).

All participants provided written informed consent prior to enrollment(IRB approval: ICDC-2022-001). Inclusion criteria: age 18-70 years; BMI 16-30 kg/m^2; normotensive (<140/90 mmHg); normoglycemic (fasting glucose <7.0 mmol/L); no history of malignancy, chronic gastrointestinal disorders. Genomic DNA were extracted from all samples within 24h after collection.

2 DNA extraction, sequencing, and quality control analysis

DNA was extracted from 180-220 mg feces using the QIAamp Fast DNA Stool Mini Kit (Qiagen #51604) following manufacturer's protocols. DNA purity was verified by spectrophotometry (NanoDrop: A260/280>1.8, A260/230>2.0) . Libraries were prepared with the TruePrep DNA Library Prep Kit (Vazyme #TD503) using 50 ng input DNA, which was enzymatically fragmented to gernerate 300-500bp insert sizes. Paired-end sequencing(2×150 bp) was performed on Illumina Hiseq platform (average output: 32.7 million reads/sample, Q30>80%). Raw data have been deposited in NCBI SRA (Accessions: SAMN44930011-SAMN44930200, SAMN41553299-SAMN41553537). For comparative analysis, public available HMP fecal microbiome data were obtained from http://www.hmpdacc.org/. Shotgun metagenomics data from 148 healthy individuals were downloaded, and all samples were obtained from healthy individuals based on project metadata, and then analyzed using the same pipeline below.

3 Bioinformatic Analysis

3.1 Data Preprocessing

Raw reads were quality-controlled with Fastp v0.23.2 with stringent parameters (--cut_right --cut_mean_quality 20 --length_required 100)(Chen et al 2018) . Host-derived DNA was removed using Kraken2 v2.1.2 (standard database) (Wood et al 2019). Metagenome-assembled genomes (MAGs) were generated by metaSPAdes v3.13.0 with optimized parameters (k-mer:21,33,55,77,99, --meta -t 32 -m 1000)(Nurk et al 2017).

3.2 Plasmid Prediction and Clustering

The analysis pipeline developed in the present study is available on GitHub (https://github.com/zhangwencdc/pGut).

Metagenomic reads were assembled using Spades v3.13.0 (Nurk et al 2017). Contigs were screened for plasmid sequences using four complementary approaches: GeNomad(Camargo et al 2024), PlasmidFinder(Carattoli and Hasman 2020), PlasmidHunter(Tian et al 2024), and an in-house developed PlasmidCircular method. To ensure high-confidence predictions, only plasmid predictions result supported by ≥2 tools were retained. Predicted plasmids were clustered with Galah(Parks et al 2020), defining clusters at thresholds of >70% alignment coverage (Align) and >97% average nucleotide identity (ANI).

Plasmid gene prediction and annotation were performed with Prokka(Seemann 2014). Antimicrobial resistance genes (ARGs) were identified by aligning all plasmid sequences against the ResFinder database(Florensa et al 2022) using Blat(Kent 2002), with positve hits defined if Align≥80% of a reference gene was covered. Plasmid structures and gene annotations were visualized using Proksee(Grant et al 2023).

4 Experimental Validation

4.1 Full-length pGut1 validation

Complete plasmid sequence was reconstructed through PCR amplification using eleven primer pairs spanning pGut1 backbone (Supplemental Table 2). Reactions

were performed with Q5 polymerase (94°C/10s, 55°C/5s, 60°C/90s, 25 cycles). Sanger sequencing (ABI 3730XL) and assembly (SeqMan v5.0) generated circular sequences.

4.2 Long-read Nanopore validation

Genomic DNA extracted from one pGut1-positive sample (20ZYI1) underwent Nanopore sequencing. Libraries were prepared using the SQK-LSK114 kit and sequenced on a GridION X5 platform (R10.4.1 flowcell, 24hr runtime).

Raw Nanopore reads were error-corrected using LoRDEC v2.9(Salmela and Rivals 2014) with Illumina short reads as reference. Corrected reads were converted to FASTA format using SeqKit fq2fa tool(Shen et al 2024) and aligned against the reference pGut1 sequence with BLAT(Kent 2002). Align covering ≥80% of the plasmid length were retained for validation. Schematic representations of the sequence alignments were generated using the NGenomeSyn tool(He et al 2023).

5 Single-cell sequencing validation

Publicly available single-cell sequencing datasets (PRJNA803937) were retrieved from NCBI. Metagenomic assembly was performed using SPAdes(Nurk et al 2017). Contigs were aligned against the pGut1 reference plasmid with BLAT(Kent 2002), retaining hits covering ≥80% of the plasmid length. Variable and backbone regions of pGut1 were extracted from positive contigs using SeqKit amplicon tool(Shen et al 2024) with primer pairs (pGut1_P11 and pGut1_P11_reverse). Primer sequences were previously validated by Sanger sequencing. Plasmid structures and gene annotations were visualized using Proksee(Grant et al 2023).

6 Large-scale Genome Screening and Evolutionary Analysis

All 2,299,771 publicly available bacterial genomes (accessed January 2025) were retrieved from NCBI. Each genome was aligned against the pGut1 reference plasmid using BLAT(Kent 2002), retaining hits covering ≥80% of the plasmid length.

Variable and backbone regions were extracted from positive genomes using SeqKit amplicon(Shen et al 2024) with validated primer pairs (pGut1_P11 and pGut1_P11_reverse). Primer sequences were previously validated by Sanger sequencing. Backbone regions from all identified pGut1 plasmids underwent multiple

sequence alignment with MAFFT(Katoh and Standley 2013). Evolutionary relationships were reconstructed using FastTree (-nt -gtr)(Price et al 2010) and visualized with iTOL(Letunic and Bork 2021). Multilocus sequence typing (MLST) and serotyping of the corresponding bacterial strains were performed using MLST(Jolley and Maiden 2010) and Seqsero2(Zhang et al 2019a), respectively.

Use of generative AI

The original draft of this manuscript was written in Chinese. The English translation of the text was assisted by the generative AI model DeepSeek (DeepSeek Company). No generative AI or large language models were used in the conceptualization, data analysis, interpretation, figure creation, or decision-making processes of the research.

Reference

Camargo AP, Roux S, Schulz F, Babinski M, Xu Y, Hu B *et al* (2024). Identification of mobile genetic elements with geNomad. *Nat Biotechnol* **42:** 1303-1312.

Carattoli A, Hasman H (2020). PlasmidFinder and In Silico pMLST: Identification and Typing of Plasmid Replicons in Whole-Genome Sequencing (WGS). *Methods Mol Biol* **2075:** 285-294.

Chen S, Zhou Y, Chen Y, Gu J (2018). fastp: an ultra-fast all-in-one FASTQ preprocessor. *Bioinformatics* **34:** i884-i890.

Florensa AF, Kaas RS, Clausen P, Aytan-Aktug D, Aarestrup FM (2022). ResFinder - an open online resource for identification of antimicrobial resistance genes in next-generation sequencing data and prediction of phenotypes from genotypes. *Microb Genom* **8**.

Grant JR, Enns E, Marinier E, Mandal A, Herman EK, Chen CY *et al* (2023). Proksee: in-depth characterization and visualization of bacterial genomes. *Nucleic Acids Res* **51:** W484-w492.

He W, Yang J, Jing Y, Xu L, Yu K, Fang X (2023). NGenomeSyn: an easy-to-use and flexible tool for publication-ready visualization of syntenic relationships across multiple genomes. *Bioinformatics* **39**.

Jolley KA, Maiden MC (2010). BIGSdb: Scalable analysis of bacterial genome variation at the population level. *Bmc Bioinformatics* **11:** 595.

Katoh K, Standley DM (2013). MAFFT multiple sequence alignment software version 7: improvements in performance and usability. *Mol Biol Evol* **30:** 772-780.

Kent WJ (2002). BLAT--the BLAST-like alignment tool. *Genome Res* **12:** 656-664.

Letunic I, Bork P (2021). Interactive Tree Of Life (iTOL) v5: an online tool for phylogenetic tree display and annotation. *Nucleic Acids Res* **49:** W293-w296.

Nurk S, Meleshko D, Korobeynikov A, Pevzner PA (2017). metaSPAdes: a new versatile metagenomic assembler. *Genome Res* **27:** 824-834.

Parks DH, Chuvochina M, Chaumeil P-A, Rinke C, Mussig AJ, Hugenholtz P (2020). A complete domain-to-species taxonomy for Bacteria and Archaea. *Nature Biotechnology* **38:** 1079-1086.

Price MN, Dehal PS, Arkin AP (2010). FastTree 2--approximately maximum-likelihood trees for large alignments. *PLoS One* **5:** e9490.

Salmela L, Rivals E (2014). LoRDEC: accurate and efficient long read error correction. *Bioinformatics* **30:** 3506-3514.

Seemann T (2014). Prokka: rapid prokaryotic genome annotation. *Bioinformatics* **30:** 2068-2069.

Shen W, Sipos B, Zhao L (2024). SeqKit2: A Swiss army knife for sequence and alignment processing. *Imeta* **3:** e191.

Tian R, Zhou J, Imanian B (2024). PlasmidHunter: accurate and fast prediction of plasmid sequences using gene content profile and machine learning. *Brief Bioinform* **25**.

Wood DE, Lu J, Langmead B (2019). Improved metagenomic analysis with Kraken 2. *Genome biology* **20:** 257.

Zhang S, Bakker HCd, Li S, Chen J, Dinsmore BA, Lane C *et al* (2019a). SeqSero2: Rapid and Improved Salmonella Serotype Determination Using Whole-Genome Sequencing Data. *Applied and Environmental Microbiology* **85:** e01746-01719.

Zhang W, Li J, Lu S, Han N, Miao J, Zhang T *et al* (2019b). Gut microbiota community characteristics and disease-related microorganism pattern in a population of healthy Chinese people. *Sci Rep* **9:** 1594.

Zhang W, Han N, Zhang T, Qiang Y, Peng X, Li X *et al* (2022). The Spatial Features and Temporal Changes in the Gut Microbiota of a Healthy Chinese Population. *Microbiol Spectr* **10:** e0131022.
